# Evaluating the psychometric properties of the Widespread Pain Index and the Symptom Severity Scale in youth with painful conditions

**DOI:** 10.1080/24740527.2019.1620097

**Published:** 2019-06-26

**Authors:** Joanne Dudeney, Emily F. Law, Alagumeena Meyyappan, Tonya M. Palermo, Jennifer A. Rabbitts

**Affiliations:** a Center for Child Health, Behavior and Development, Seattle Children’s Research Institute, Seattle, Washington, USA; b School of Women’s and Children’s Health, Faculty of Medicine, University of New South Wales, Sydney, New South Wales, Australia; c Department of Anesthesiology and Pain Medicine, University of Washington School of Medicine, Seattle, Washington, USA; d Center for Clinical and Translational Research, Seattle Children’s Research Institute, Seattle, Washington, USA

**Keywords:** chronic pain, child, centralized pain, pain distribution, pain location, widespread pain

## Abstract

**Background**: Assessing features of centralized pain may prove to be clinically meaningful in pediatric populations. However, we are currently limited by the lack of validated pediatric measures.

**Aim**: We examined the psychometric properties of the Widespread Pain Index (WPI) and Symptom Severity (SS) scale to assess features of centralized pain in youth with painful conditions from three clinical samples: (1) musculoskeletal surgery, (2) headache, and (3) chronic pain.

**Methods**: Participants were 240 youth aged 10 to 18 years (*M*_age_ = 14.8, SD = 1.9) who completed the WPI and SS scale. Subsets of participants also completed additional measures of pain region, pain intensity, quality of life, pain interference, and physical function.

**Results**: Increased features of centralized pain by age were seen for the WPI (*r* = 0.27, *P* < 0.01) and SS scale (*r* = 0.29, *P* < 0.01). Expected differences in sex were seen for the WPI (sex: t_132_ = −3.62, *P* < 0.01) but not the SS scale (sex: *t*_223_ = −1.73, *P* = 0.09). Reliability for the SS scale was adequate (α = 0.70). Construct validity was demonstrated through relationships between the WPI and pain regions (*r* = 0.57, *P* < 0.01) and between the SS scale and quality of life (*r* = −0.59, *P* < 0.01) and pain interference (*r* = 0.56, *P* < 0.01). Criterion validity was demonstrated by differences on the WPI between the surgery sample and the headache and chronic pain samples (F_2,237_ = 17.55, *P* < 0.001). Comprehension of the SS scale items was problematic for some youth.

**Conclusions**: The WPI showed adequate psychometric properties in youth; however, the SS scale may need to be modified. Our findings support the need to develop psychometrically sound instruments for comprehensive assessment of pain in pediatric samples.

## RÉSUMÉ

**Contexte**: L’évaluation des caractéristiques de la douleur centralisée peut se révéler significative cliniquement chez les populations pédiatriques. Toutefois, nous sommes actuellement limités par le manque de mesures pédiatriques validées.

**Objectif**: Nous avons étudié les propriétés psychométriques de l’Index de douleurs généralisées (WPI) et de l’Échelle de sévérité des symptômes (SS), afin d’évaluer les caractéristiques de la douleur centralisée chez les jeunes atteints d’affections douloureuses issus de trois échantillons cliniques : (1) chirurgie musculosquelettique ; (2) mal de tête, et (3) douleur chronique.

**Méthodes**: Les participants étaient 240 jeunes âgés de 10 à 18 ans (Âge moyen = 14,8, É.-T. = 1,9), qui avaient répondu au WPI et à l’Échelle SS. Des sous-ensembles de participants ont également répondu à d’autres questionnaires portant sur l’emplacement de la douleur, l’intensité de la douleur, la qualité de vie, l’interférence de la douleur et la fonction physique.

**Résultats**: Une augmentation des caractéristiques de la douleur selon âge a été observée pour le WPI (r = 0,27, *p* < 0,01) et pour l’Échelle SS (sexe : t_223_ = −1,73, *p* = 0,09). Les différences attendues entre les sexes ont été observées pour le WPI (sexe : t_132_ = −3,62, *p* < 0,01), mais pas pour l’Échelle SS (sex : t_223_ = −1,73, *p* = 0,09). La fiabilité de l’échelle SS était adéquate (α = 0,70). La validité du construit a été démontrée par la relation entre le WPI et l’emplacement de la douleur (*r* = .57, *p* < .01), et entre l’Échelle SS et la qualité de vie (*r* = −.59, *p* < .01) et l’interférence de la douleur *r* = .56, *p* < .01). La validité des critères a été démontrée par les différences pour le WPI entre l’échantillon ayant subi une chirurgie et les échantillons de patients souffrant de mal de tête et de douleur chronique (F_2,237_ = 17,55, *p* < 0,001). La compréhension des énoncés de l’Échelle SS était problématique pour certains jeunes.

**Conclusions**: Le WPI a démontré des propriétés psychométriques adéquates chez les jeunes; toutefois, l’Échelle SS pourrait devoir être modifiée. Nos constatations viennent corroborer la necessité d’élaborer des instruments solides sur le plan psychométrique, permettant d’évaluer globalement la douleur chez les échantillons pédiatriques.

## Introduction

Pain can modify the central nervous system, so that an individual experiences more pain with less provocation. This process is called “central sensitization” because it involves heightened responsiveness of pain signals in the brain and the spinal cord,^1^^,^^2^ which increases sensitivity to pain. Clinical studies have demonstrated a number of chronic conditions (e.g., fibromyalgia, rheumatologic diseases, chronic pancreatitis, chronic pelvic pain) in which heightened pain responsiveness and greater spatial extent of pain (thought to be phenomena of central sensitization) are part of the pain phenotype.^3–7^

Methods such as quantitative sensory testing and brain imaging technologies have typically been used to study mechanisms of central sensitization.^8^ Research using these modalities has demonstrated heightened pain sensitivity, increased pain facilitation, diminished pain inhibition, and alteration in brain function and structure as markers of central sensitization in adults.^9–11^ Similarly, in youth, quantitative sensory testing has identified greater sensitivity to pain for those with conditions such as juvenile idiopathic arthritis, fibromyalgia, and functional abdominal pain, indicating that central sensitization is also part of the pain phenotype in pediatric pain conditions.^12–14^ Recently, researchers have utilized self-report to investigate clinical features of centralized pain, including spatial distribution of pain and cognitive, emotional, and physical symptoms.^15,16^ Although self-report is an indirect tool to assess central sensitization, it remains the gold standard in pain assessment.^8^ Indeed, comprehensive assessment of pain, which includes assessment of bodily distribution of pain, is needed to accurately classify both acute and chronic pain.^17,18^ Valid and reliable self-report measures are essential for characterizing pain features in the clinical setting, particularly in pediatric populations, where objective testing (e.g., quantitative sensory testing or brain imaging) may not be available or feasible. However, a major limiting factor in assessing features of centralized pain in youth via self-report is the lack of validated pediatric measures.

The Widespread Pain Index (WPI) and Symptom Severity (SS) scale is a self-report measure assessing pain distribution (WPI) and the severity of six symptoms, including fatigue, memory difficulties, tiredness, headache, abdominal pain, and depression (SS scale).^19,20^ The WPI and SS scale was originally developed to classify fibromyalgia in adults using the adapted 2010 American College of Rheumatology fibromyalgia survey criteria^21–23^; however, the combined measure has since been utilized more widely to assess degree of widespread body pain and centralized pain features (e.g., cognitive, emotional, and physical symptoms) in studies of general chronic pain conditions,^24,25^ and surgical samples.^16,26^ The few available studies examining features of centralized pain in pediatric populations, as measured by self-report, have used the combined WPI and SS scale.^27–29^ However, the psychometric properties of the WPI and SS scale have not been evaluated in pediatric populations to understand whether the measure is reliable and valid, outside of assessing diagnostic utility in youth with fibromyalgia.^29^

To address this gap, the aim of the current study was to assess the psychometric properties of the WPI and SS scale in three clinical samples of youth (musculoskeletal surgery, headache, chronic pain) that would be expected to differ in the degree of centralized pain features based on the location, severity, and chronicity of pain. Because the WPI and SS scale was not developed for use in pediatric populations, we were also interested in assessing comprehension of instructions and items. First, based on research showing higher incidence of centralized pain conditions (e.g., widespread pain and juvenile fibromyalgia) in females compared to males and adolescents compared to children,^30,31^ we expected that higher scores on the WPI and higher SS scales would be shown for females compared to males and older adolescents compared to younger adolescents. Second, we hypothesized that reliability for the SS scale would be demonstrated through strong internal consistency and interitem and item total correlations. Third, based on previous research showing strong relationships between features of centralized pain and pain and quality of life outcomes,^28,32,33^ we expected to demonstrate construct validity via strong associations between (1) the WPI score and number of pain regions, (2) the SS scale and measures of quality of life and pain interference, and (3) the total score and measures of pain (regions, intensity) and function (quality of life, pain interference, physical function). Fourth, we hypothesized that criterion validity would be demonstrated through significant differences in pain features between clinical samples, which theoretically should have differing levels of features of centralized pain. Specifically, we expected that higher scores on the WPI and higher SS scales would be found for those in the chronic pain and headache groups, when compared to those from the musculoskeletal surgery group, based on prior research demonstrating both persistent pain in multiple locations and heightened pain sensitivity in chronic headache and chronic pain conditions.^34–36^ Finally, we hypothesized that youth would demonstrate adequate comprehension of the WPI and SS scales, based on individual interviews.

## Materials and methods

### Participants

Participants included 240 youth, 10 to 18 years of age, enrolled in one of three studies at a tertiary children’s hospital in the Pacific Northwest United States. The participants included (1) 89 youth with musculoskeletal conditions scheduled to undergo major musculoskeletal surgery (spinal *n* = 62; pectus *n* = 22; other *n* = 3), (2) 56 youth with frequent or chronic headache as a primary pain complaint (i.e., at least eight or more headache days a month for at least 3 months) and pain in at least one other location, and (3) 97 youth presenting for evaluation of chronic pain; that is, recurrent or persistent pain experienced for at least 3 months (musculoskeletal *n* = 51; abdominal *n* = 19; headache *n* = 19; other *n* = 8). In recruiting each of the three samples (musculoskeletal surgery, headache, chronic pain), research teams identified potentially eligible participants from the surgery clinic and operating room schedules, after new patient evaluations in a pediatric neurology clinic or from the community, and after new patient evaluations in an interdisciplinary pediatric pain clinic, respectively. Data reported in the current study were collected during the baseline assessment (pretreatment) phase of each study. Exclusion criteria were consistent across studies and included (1) the presence of a serious medical comorbidity (e.g., cancer), (2) a severe developmental delay, or (3) the youth or parent was non-English speaking.

### Procedures

The local institutional review board approved all procedures for the three studies. Across studies, research assistants screened potentially eligible youth via telephone; provided eligible families with electronic copies of the consent, assent, and HIPPA forms; and obtained verbal consent and assent via telephone prior to starting any study procedures. All participants completed survey measures online, via a secure Research Electronic Data Capture link.^37^ A subset of participants (*n* = 70) from the surgery sample completed a pain region body map on paper. Participants received gift cards for completion of online assessments.

Research staff (MM, JD) individually contacted a sample of participants and conducted a brief telephone interview to assess comprehension of the WPI and SS scales. We employed convenience sampling, in which we only contacted participants for the comprehension interview if they had completed the measure within the past 10 weeks; thus, we were unable to conduct an equal number of interviews across the samples. Eleven participants completed the interview: six from the surgery sample, four from the headache sample, and two from the chronic pain sample. We found no demographic differences (e.g., age, sex, ethnicity, parent education) between the participants interviewed and the full sample (all *P*s > 0.05).

### Measures

#### Widespread Pain Index and Symptom Severity Scale

The WPI and SS scale is a 27-item self-report measure used to assess bodily distribution of pain and to specifically quantify the degree of widespread body pain and assess for centralized pain features (e.g., cognitive, emotional, and physical symptoms).^19,20^ It consists of two scales, one assessing pain distribution from focal to widespread (WPI) and the other assessing the presence and severity of symptoms associated with centralized pain (SS scale). The WPI assesses presence of pain in 19 designated body locations over the past 7 days (e.g., neck, right upper arm, left lower leg). Each location is equal to a score of 1. Items are summed to yield a total score, with higher scores indicating greater widespread pain. The six-item SS scale assesses (1) presence of clinical symptoms (lower abdomen pain, headache, depression) over the past 6 months and (2) the severity of cognitive symptoms (fatigue, trouble thinking or remembering, waking up tired/unrefreshed) over the past 7 days. Individuals are asked about whether they experience these symptoms generally, not specifically related to or as a consequence of their pain. The presence of a clinical symptom is equal to a score of 1. The severity of cognitive symptoms is scored on a 4-point scale where 0 indicates *no problem* and 3 indicates *severe problem*. Scores are calculated by summing items, with higher scores (out of a maximum score of 12) indicating greater symptom severity. The WPI and SS scales can be combined to create a total score (range 0–31), with higher scores indicating greater centralized pain features. The measure includes two additional questions that do not contribute to the overall score, the first assessing the chronicity of symptoms and the second determining whether symptoms are due to a pre-existing disorder. All participants completed the WPI and SS scale.

#### Body diagram

A previously validated self-report body diagram for youth was used to assess pain locations over the past 7 days in a subset of youth in the surgery sample.^38^ Youth indicated locations where they experienced aches or pain by drawing an “X” on a body outline showing the front and back of the body. The pain locations were coded into five regions in accordance with Jones et al.^39^ and based on the 1990 American College of Rheumatology definition of widespread pain: left side of body, right side of body, above waist (head, neck, arms, hands, upper body, chest, abdomen), below waist (lower abdomen/pelvis, low back, buttocks, legs, feet), and axial (spine, chest, or back), with the presence of pain in a region equal to a score of 1, for a total score out of 5.^39,40^ A single pain location could be coded into two regions (e.g., axial and below waist for low back pain). A total pain region score is calculated by summing the number of regions coded and categorizing the score into one of the following: two or fewer regions, three regions, four regions, and five regions. This coding scheme has been used in prior studies of pain distribution in youth.^41^

#### Pediatric Quality of Life Inventory

The Pediatric Quality of Life Inventory, Short Form version 4.0 (PedsQL) was used to assess health-related quality of life over the previous 4 weeks.^42^ The PedsQL contains 15 items; 10 assess the core domain of psychosocial functioning (e.g., “I feel sad or blue”) and 5 assess the core domain of physical function (e.g., “It’s hard for me to do sports activity or exercise”). Items are scored on a 5-point Likert scale, where 0 indicates *never* and 4 indicates *almost always*. The summary scores for each core domain are totaled and converted to a 0 to 100 point range, with higher scores indicating better health-related quality of life. The PedsQL has shown reliability and criterion and construct validity in healthy, chronically ill, and acutely ill youth.^42^ This measure was completed by the surgery and headache samples. Internal consistency was excellent for the physical health domain (α = 0.86) and the psychosocial health domain (α = 0.85).

Patient-Reported Outcomes Measurement Information System v2.0 Pediatric Profile–25

The sample with chronic pain completed the Patient-Reported Outcomes Measurement Information System Pediatric Profile instrument, a collection of short forms containing a total of 25 items from seven domains (pain intensity, pain interference, anxiety, depressive symptoms, fatigue, peer relationships, and physical function mobility). The measure assesses physical and psychosocial health and well-being in youth over the preceding 7 days. In the current study, we used the domains pain interference, physical function mobility, and pain intensity. The pain interference and physical function mobility domains both include four items. Items for pain interference are scored on a 5-point Likert scale where 1 indicates *never* and 5 indicates *almost always*. Items for physical function mobility are scored on a 5-point Likert scale where 1 indicates *with no trouble* and 5 indicates *not able to do*. For both domains, total raw scores are transformed into standardized *T*-scores for analyses. The *T*-score distribution has a mean of 50 (SD ± 10), with scores of more than one standard deviation higher or lower than the mean considered clinically meaningful. The Patient-Reported Outcomes Measurement Information System profile has been used in youth experiencing chronic pain.^43,44^ Internal consistency for the domains ranged from good (α = 0.75) to excellent (α = 0.93).

The pain intensity domain includes a single question, “How bad was your pain on average?” The question is scored using an 11-point numerical rating scale, with 0 indicating *no pain* and 10 indicating *worst pain possible*. Because this is a single item, it precludes reliability analysis.

#### Interviews to assess comprehension of the WPI and SS scale

A subset of participants (*n* = 11) were contacted by phone for comprehension interviews and asked to re-complete the WPI and SS online via Research Electronic Data Capture and to verbally indicate when they finished. Participants were then asked questions assessing comprehension of instructions and item comprehension (e.g., “What is this question asking?”; “In this question, what does ‘fatigue’ mean?”). Responses were coded dichotomously (yes/no) as to whether participants comprehended instructions and specific items. Adequate comprehension of instructions was defined as the ability to (1) describe in their own words what they needed to do to complete each item and (2) explain the meaning of the response options for all items (e.g., “slight or mild problem”). Adequate item comprehension was defined as the ability to interpret a series of five key words in the items (fatigue, widespread, intermittent, depression, disorder) identified by the research team as possibly exceeding the expected reading level of the sample.

### Data analysis plan

All analyses were conducted with SPSS version 21.^45^ Missing data were minimal (8%). Youth in the surgery sample had more missing data because questionnaire items were presented as optional for this study only but were required for the headache and chronic pain samples. We found no demographic differences (e.g., age, sex, ethnicity, parent education) between the participants with versus without missing data. We therefore deemed that data were missing completely at random and used all available data in the analyses. We considered results statistically significant at *P* < 0.05. We report partial eta squared where appropriate, which is interpreted as follows: 0.01 = a small effect size, 0.06 = a medium effect size, and 0.14 = a large effect size.^46^ For correlational analyses, the size of *r* is interpreted as 0.1 =  small, 0.3 =  medium, and 0.5 =  large.^47^

The SS scale includes one item asking whether participants experienced a headache over the past 6 months. Given that this item could inflate results for the headache group, we ran the analyses twice: once with all items included and a second time with the headache item removed for the headache sample. Removal of the item did not change the magnitude or direction of the results; thus, we have presented the analyses with the headache item included.

#### Participant characteristics

We computed descriptive statistics for the sample demographics. We conducted *t*-tests to assess whether the WPI, SS scale, and total scores differed by sex and Pearson correlational analyses to determine differences by age. Based on related research into widespread pain and juvenile fibromyalgia, we expected higher WPI, SS scale, and total scores for older youth and females.^30,31^

#### Reliability

We assessed reliability of the SS scale through interitem and item total Pearson correlations and we assessed internal consistency (Cronbach’s alpha) of the three SS scale items scored on a continuous scale for the full sample. The WPI locations and the remaining three SS scale items are scored as dichotomous (yes/no) variables, which precluded analysis of internal consistency. Clark and Watson^48^ recommend mean interitem correlations within the range of 0.15 to 0.20 for scales measuring broad characteristics, such as headache, depression, and abdominal pain, and between 0.40 and 0.50 for scales measuring narrower characteristics, such as the cognitive symptom severity construct (fatigue, memory, tiredness). For item total correlations, a recommended cutoff point for retaining items is between *r* = 0.30^48^ and a more conservative *r* = 0.40.^49^

#### Validity

##### Construct validity

We conducted Pearson correlations to assess the relationship between the WPI, SS scale, and total scores with other measures of number of pain locations and child functioning. The domains included in the analyses were pain region, pain intensity, psychosocial health, physical health, pain interference, and physical function mobility.

##### Criterion validity

We investigated the validity of the measure to discriminate between groups that should, theoretically, have differing levels of features of centralized pain. Because we included three samples, we conducted analyses of variance (ANOVAs) and Bonferroni post hoc tests to compare WPI and SS scale and determine whether the measure showed expected differences between the samples. We expected higher WPI, SS scale, and total scores for youth with persistent pain conditions (chronic pain and headache groups).

#### Comprehension

Descriptive statistics were used to summarize the number of participants who demonstrated adequate comprehension of instructions and adequate item comprehension.

## Results

### Participant characteristics

Participant characteristics are presented in Table 1. There were no significant differences between the surgery, headache, or chronic pain samples on any of the demographic variables. There was a significant difference in pain intensity between the three samples; that is, youth with chronic pain reported significantly higher pain intensity than both other samples and youth with headache reported significantly higher pain intensity than the surgery sample. Item-level summary statistics for the SS scale are presented in Table 2.

**Table 1.** Participant demographic characteristics (*n* = 240).

	Surgery sample	Headache sample	Chronic pain sample	Whole sample	Demographic differences
	*n* (%)^a^	*n* (%)	*n* (%)	*n* (%)	
*n*	87	56	97	240	
Age, *M* (SD)	14.9 (1.9)	14.8 (1.9)	14.7 (1.8)	14.8 (1.9)	F_2,237_ = 0.22, *P* = 0.80, η^2^ = 0.002^b^
Sex, female	60 (69.0)	40 (71.4)	80 (82.5)	180 (75.0)	χ^2^_2_ = 4.96, *P* = 0.08^c^
Race White African American Asian Other Not reported	66 (75.9) 5 (5.7) 3 (3.4) 6 (6.8) 7 (8.0)	46 (82.1) 0 2 (3.6) 6 (10.7) 2 (3.6)	79 (81.4) 2 (2.1) 3 (3.1) 12 (12.3) 1 (1.0)	191 (79.6) 7 (2.9) 8 (3.3) 24 (10) 10 (4.2)	χ^2^_10_ = 8.75, *P* = 0.56^c^
Ethnicity Hispanic Non-Hispanic Unknown Not reported	7 (8.0) 73 (83.9) 1 (1.1) 6 (6.9)	4 (7.1) 49 (87.5) 3 (5.4) 0	5 (5.2) 86 (88.7) 5 (5.2) 1 (1.0)	16 (6.7) 208 (86.7) 9 (3.8) 7 (2.9)	χ^2^ = 3.01, *P* = 0.56^c^
Parent education High school or less Vocational school/some college College Graduate/professional school Not reported	14 (16.1) 21 (24.1) 35 (40.2) 13 (14.9) 4 (4.6)	2 (3.6) 16 (28.6) 24 (42.9) 14 (25.0) 0	12 (12.4) 29 (29.9) 35 (36.1) 18 (18.6) 3 (3.1)	28 (11.7) 66 (27.5) 94 (39.2) 45 (18.8) 7 (2.9)	χ^2^_6_ = 7.37, *P* = 0.29^c^
Pain intensity, *M* (SD)	4.2 (1.5)	5.0 (1.9)	5.8 (1.8)	5.1 (1.8)	F_2,224_* = 18.90, P* <0.01, η^2^ = 0.15^d^

^a^All percentages may not equal 100 due to rounding.

^b^ANOVA (Mean age × Group)

^c^Chi-square test (Dependent categorical variable [e.g., Sex] × Group).

^d^ANOVA (Mean pain intensity × Group).

ANOVA = analysis of variance.

As hypothesized, females had higher WPI and total scores than males, indicating differences in widespread pain and overall features of centralized pain by sex (see Table 3). Contrary to our expectation, we did not identify statistically significant differences by sex for the SS scale. As hypothesized, we found greater WPI, SS scale, and total scores as youth increased in age, indicating that older children had greater features of pain centralization (WPI: *r* = 0.27, SS scale: *r* = 0.29, and total score: *r* = 0.32; all *P*s *≤* 0.01).

**Table 2.** Symptom Severity scale item-level descriptives.

		Mean	SD	Range^a^
Question 1: For each symptom listed below, use the following scale to indicate the severity of the symptom during the past 7 days.				
Fatigue		1.2	1.0	0–3
Trouble thinking or remembering		0.9	0.9	0–3
Waking up tired		1.6	0.9	0–3
Question 2: During the past 6 months have you had any of the following symptoms?				
		Yes, *n* (%)		
Pain or cramps in lower abdomen		131 (54.6)		
Depression		90 (37.5)		
Headaches		190 (79.82)		

^a^Range of participant responses (scale range 0–3); higher scores indicate greater symptom severity.

**Table 3.** Analyses of the WPI, SS scale, and total scores by sex.

	Male^a^	Female	Mean difference (SE)^b^	95% CI
WPI, *M* (SD)	2.9 (3.4)	4.9 (4.5)	−2.02 (0.56)	−3.12, −0.91*
SS scale, *M* (SD)	5.0 (2.5)	5.7 (2.8)	−0.70 (0.41)	−1.55, 0.15
Total score, *M* (SD)	8.1 (4.9)	10.8 (6.3)	−2.66 (0.82)	−4.29, −1.03*

^a^Male *n* = 60, female *n* = 180.

^b^Mean difference was calculated as the first variable minus the second; thus, negative values indicate greater scores in females.

*P < 0.05.

WPI = Widespread Pain Index; SS = Symptom Severity; CI = confidence interval.

### Reliability

We assessed reliability of the SS scale through internal consistency and interitem and item total correlations for the full sample (see Table 4). Internal consistency for cognitive symptoms was adequate (α = 0.70). Interitem analyses revealed small to large correlations with a range of *r* = 0.13 to 0.53. As expected from the literature,^48^ smaller correlations were seen between headache, depression, and abdominal pain items, and larger correlations were seen between the cognitive symptoms items. Item total correlations were medium to large with a range of *r* = 0.44 to 0.79. As hypothesized, all item total correlations were above the recommended cutoff point of *r* = 0.40,^50^ supporting reliability of the SS scale.

**Table 4.** Symptom Severity scale interitem and item total Pearson (*r*) correlations.

	1	2	3	4	5	6
1. Fatigue						
2. Trouble thinking or remembering	0.37**					
3. Walking up tired (unrefreshed)	0.53**	0.42**				
4. Pain or cramps in the lower abdomen	0.19**	0.22**	0.23**			
5. Depression	0.28**	0.22**	0.29**	0.18**		
6. Headache	0.22**	0.25**	0.25**	0.21**	0.14*	
7. Total Symptom Severity scale	0.77**	0.71**	0.79**	0.45**	0.50**	0.44**

*P < 0.05. **P < 0.01.

### Validity

#### Construct validity

To assess construct validity, we first evaluated associations between the WPI score and a body diagram to assess pain regions. As hypothesized, we found that a greater number of pain locations on the WPI was associated with a greater number of pain regions on the body diagram (*r* = 0.57, *P* < 0.01), and this was a large association.

Next, we evaluated associations between the SS scale and previously validated youth self-report measures of quality of life and pain interference. As expected, higher scores on the SS scale were associated with greater pain interference (*r* = 0.56, *P* < 0.01), lower psychosocial quality of life (*r* = −0.59, *P* < 0.01), and lower physical quality of life (*r* = −0.36, *P* < 0.01). These associations were moderate to large.

Third, we evaluated associations between the total score (WPI and SS scale combined) and measures of pain and function. As hypothesized, the total score was associated with a greater number of pain regions reported on the body diagram (*r* = 0.46, *P* < 0.01), higher pain intensity (*r* = 0.36, *P* < 0.01), and higher level of pain interference (*r* = 0.33, *P* < 0.01), as well as lower psychosocial quality of life (*r* = −0.51, *P* < 0.01), lower physical quality of life (*r* = −0.32, *P* < 0.01), and poorer physical function (*r* = −0.30, *P* < 0.01). These were moderate to large associations.

#### Criterion validity

Consistent with our hypothesis, we identified significant differences between our clinical samples on the WPI, SS scale, and total scores (see Table 5). Bonferroni post hoc tests revealed that the surgery sample had significantly lower WPI, SS scale, and total scores compared to the headache and chronic pain samples. As expected, we did not identify statistically significant differences between the headache and chronic pain groups on the WPI, SS scale, or total scores (all *P*s > 0.05), indicating that both groups of youth with persistent pain had similar scores.

**Table 5.** Analyses of the WPI, SS scale, and total scores by clinical population to assess criterion validity.

					Bonferroni post hoc analyses	
	Surgery (1)	Headache (2)	Chronic pain (3)	Group differences^a^	Mean difference (SE)^b^	95% CI
WPI, *M* (SD)	2.4 (2.0)	4.6 (3.8)	6.0 (5.4)	F_2,237_ = 17.55, *P* < 0.001, η^2^ = 0.13	1:2 = −2.20 (0.70) 1:3 = −3.56 (0.60)	−3.89, −0.51* −5.02, −2.11*
SS scale, *M* (SD)	3.6 (2.5)	6.6 (2.1)	6.3 (2.6)	F_2,222_ = 33.09, *P* < 0.001, η^2^ = 0.23	1:2 = −3.04 (0.44) 1:3 = −2.72 (0.38)	−4.09, −1.98* −3.63, −1.80*
Total score, *M* (SD)	6.5 (3.5)	11.2 (4.9)	12.3 (7.0)	F_2,222_ = 23.74, *P* < 0.001, η^2^ = 0.18	1:2 = −4.73 (0.99) 1:3 = −5.78 (0.86)	−7.11, −2.35* −7.86, −3.69*

^a^ANOVA (Mean score × Group).

^b^Mean difference was calculated as the first variable minus the second; thus, negative values indicate greater scores in headache and chronic pain samples.

*P < 0.001.

WPI = Widespread Pain Index; SS = Symptom Severity; CI = confidence interval; ANOVA = analysis of variance.

### Understanding of the WPI and SS scale

#### Comprehension of instructions

All 11 participants interviewed (*M*_age_ = 15.8 years, range = 12.5–17.6, SD = 1.5; 73% female) met our criteria for adequate comprehension of the instructions for completing the WPI and SS scale.

#### Item comprehension

We found that participants were unable to demonstrate adequate comprehension of the following words from the SS scale: “depression” (incorrectly defined by three participants), “intermittent” (incorrectly defined by six participants), “fatigue” (unable to be defined by six participants), “widespread” (incorrectly defined by four participants), and “disorder” (unable to be defined by two participants). Participants either provided an incorrect interpretation of the above words or stated that they did not know what the word meant.

## Discussion

The present study evaluated the psychometric properties of the WPI and SS scale in three pediatric samples with painful conditions. The SS scale showed reliability through adequate internal consistency and interitem and item total correlations. The measure showed good construct validity through expected relationships between the WPI and a body diagram and between the SS scale and measures of quality of life and pain interference. It also showed good criterion validity, with the WPI and SS scale discriminating between clinical groups hypothesized to have differing features of centralized pain. In terms of expected demographic differences, only the WPI performed as hypothesized, with older adolescents and females presenting with a higher number of pain locations, as assessed by the WPI. On the contrary, the SS scale did not demonstrate expected differences in centralized pain symptoms by sex. Further, comprehension of key items on the SS scale was problematic for some youth.

Several factors may have contributed to the SS scale performing contrary to hypotheses. The WPI and SS scale was originally developed to classify fibromyalgia in adults and, as such, the SS scale assesses the specific symptoms associated with adult fibromyalgia. Research shows that youth with juvenile fibromyalgia present with comorbid and related symptoms that are less pronounced than those reported by adults.^30^ In addition, there may be distinct clinical characteristics associated with centralized pain in youth that the measure does not assess. For example, anxiety/tension is associated with a number of chronic pain conditions in youth.^51–53^ Difficulties with item comprehension may also have contributed to the performance of the SS scale. The SS scale does not include instructions on whether questions should be answered in relation to pain or in general, nor does it account for whether symptoms are a consequence of pain or a symptom of a comorbid condition (e.g., increased fatigue due to depression). Additionally, of concern, youth across the age range of 12 to 17 years demonstrated poor understanding of key terms used to assess symptoms (e.g., fatigue, depression). Similar difficulties with readability of the SS scale were found by Ting and colleagues^29^ when they examined diagnostic accuracy for fibromyalgia of the earlier 2010 version of the WPI and SS scale. Ting et al.^29^ recommended that problematic items might need to be amended for pediatric samples. However, we propose that further development of relevant items for assessment of symptoms associated with centralized pain in youth is needed.

Another potential limitation with applying this measure to populations other than those with fibromyalgia to assess features of centralized pain is that the WPI currently only includes pain locations relevant to fibromyalgia. The majority of our sample endorsed headaches on the SS scale, suggesting that this as a relevant pain location; however, the WPI does not currently include head pain. Since the development of the WPI (and our data collection commenced), a new measure of pain locations has been developed and is currently being used in conjunction with the SS scale to assess features of centralized pain in adult populations. The Michigan Body Map (MBM) includes the 19 areas from the WPI and another 16 locations (e.g., head, jaw, knee, ankle), allowing for broader research and greater clinical utility.^54^ It is similar to the WPI in that it is a continuous measure that allows for the spatial distribution of pain to be quantified. The MBM has shown good reliability when combined with the SS scale in adults,^55^ adequate validity to measure pain distribution, and good convergent validity with functional measures and was found to be preferable to the WPI by a sample of adults with pain.^54^ Thus, this may be a promising comprehensive measure to assess the spatial distribution of pain in youth as well. However, it is essential that the MBM undergo psychometric evaluation in pediatric samples before application to this population.

Relationships found in the present study between the WPI and SS scale and functional measures including physical and psychosocial health and pain interference support the notion that experiencing higher features of centralized pain can negatively affect important life domains. Prior work has examined the relationship between pain distribution, as indicated on body diagrams, and health and functional outcomes in youth in cross-sectional studies. In adolescents undergoing surgery, as well as those with acute and chronic pain conditions, widespread pain distribution was associated with poorer health-related quality of life and psychosocial health,^41^ greater school impairment,^56^ and reduced sleep quality.^57^ In a sample of adolescents with physical disabilities, greater pain distribution was associated with increased disability and decreased psychological function.^58^ Similarly, in youth with sickle cell disease, widespread pain was associated with increased pain intensity and burden, greater functional disability, impaired mood, and poorer quality of life.^32^ In a recent pediatric study in patients with idiopathic scoliosis,^28^ approximately one third of youth presented with pain profiles characterized by features of centralized pain (e.g., increased widespread pain, affective symptoms, fatigue). Following surgery, youth with more features of centralized pain reported higher acute pain intensity compared to those with fewer centralized pain features. In the longer term, youth with greater features of centralized pain reported higher chronic pain intensity, pain interference, and opioid use at 6 months following surgery.^28^ Together these studies suggest a potential longitudinal influence of central sensitization on pain outcomes, highlighting the importance of assessment of features of centralized pain in youth with painful conditions.

### Future research and clinical implications

Pain features of location and spatial distribution are critical components of the ACTTION-American Pain Society Pain Taxonomy multidimensional framework, recommended for classifying both acute and chronic pain conditions, for application in research and clinical practice.^17,18^ The ACTTION-American Pain Society Pain Taxonomy distinguishes spatial distribution of pain as a distinct dimension from pain severity (intensity). Yet, in children and adolescents, much research attention has focused on measuring pain intensity.^59^ A critical barrier is the availability of valid and reliable measures to assess broader pain features, and the present study takes an important step by evaluating a measure of features of centralized pain in youth. In the clinical context, the WPI can be used to assess pain location and distribution, which should be interpreted alongside a physical exam, which includes assessment of sensory changes (i.e., allodynia and hyperalgesia). Comprehensive pain assessment provides critical information to inform a mechanisms-based approach to pain classification.^8^ Research will be needed to guide incorporation of centralized pain features in classification of acute and chronic pain in youth to guide management.

### Limitations

The current study has several limitations. First, though inclusion of multiple clinical samples is a strength of the study, the clinical subgroups are small and the same data were not available for all youth. For example, only a subset of the surgery sample completed the body map, which was used in the analysis of construct validity of the WPI. Second, our sample lacked demographic and geographic diversity. Participants were predominately female and Caucasian, and all resided in the Pacific Northwest United States. Though our demographic characteristics are similar to those of other youth with painful conditions,^60^ as well as those who present for pediatric spinal surgery,^28^ they are not representative of the pediatric population overall. Third, we investigated associations between the WPI and SS scale and pain regions, intensity, and interference; however, other pain characteristics not captured, such as duration, could be important in assessing whether features of centralized pain develop over time (e.g., those with longer pain duration may have pain in more locations and greater associated symptoms). Fourth, our interviews were limited by a small sample size and unequal distribution across groups. However, the demographics of the sample interviewed were congruous with those of the full sample; thus, we expect limited deviation from our presented results with increased sample sizes. Finally, assessments of reliability were limited. We were not able to conduct test–retest reliability to assess the stability of the WPI and SS scale over time.

### Conclusions

The WPI and SS scale assess the spatial distribution of pain and the severity of clinical symptoms associated with centralized pain. In the current study, the WPI showed sound psychometric properties in youth with painful conditions, with expected demographic differences, good construct and criterion validity, and comprehension by youth. However, the SS scale demonstrated issues with comprehension and was unable to demonstrate all expected differences between demographic groups. Our findings support the need for further work in developing psychometrically sound instruments for comprehensive assessment of pain in pediatric samples.
